# Retraction Note: The predictive value of BTG1 for the response of newly diagnosed acute myeloid leukemia to decitabine

**DOI:** 10.1186/s13148-024-01715-w

**Published:** 2024-08-01

**Authors:** Yi Li, Xia Mao, Mengyuan Li, Li Li, Xiwen Tong, Lifang Huang

**Affiliations:** 1 Renmin Hospital of Wuhan University, Wuhan, China; 2 Department of Hematology, Tongji Hospital, Tongji Medical College, Huazhong University of Science and Technology, 1095 Jie-Fang Avenue, Wuhan, 430030 Hubei China; 3 Xinqiao Hospital of Army Medical University, Chongqing, China

**Retraction Note: Clinical Epigenetics (2024) 16:16** 10.1186/s13148-024-01627-9

The Editor-in-Chief has retracted this article at the request of the corresponding author Lifang Huang because the authors did not have permission to publish the data reported. Lifang Huang, Yi Li and Xwen Tong agree with this retraction. Xia Mao, Mengyuan Li and Li Li have not responded to correspondence from the Publisher about this retraction.

